# Drivers of youth mental health and wellbeing: a large-scale cross-sectional study in Morocco

**DOI:** 10.1136/bmjopen-2025-110683

**Published:** 2026-06-09

**Authors:** Jihad Bnimoussa, Oumnia Bouaddi, Imad El badisy, Mohamed Khalis

**Affiliations:** 1 InspireCorp, Rabat, Morocco; 2 Department of Public Health and Clinical Research, Mohammed VI Center for Research and Innovation, Rabat, Morocco; 3 Mohammed VI International School of Public Health, Mohammed VI University of Sciences and Health (UM6SS), Casablanca, Morocco; 4 AI and Data Science Service, Mohammed VI Center for Research and Innovation, Rabat, Morocco; 5 Higher Institute of Nursing Professions and Health Techniques, Ministry of Health and Social Protection, Rabat, Morocco

**Keywords:** MENTAL HEALTH, Adolescent, PUBLIC HEALTH

## Abstract

**Abstract:**

**Objectives:**

This study aimed to describe the factors influencing mental health and wellbeing from the perspective of Moroccan youth.

**Design:**

This is a descriptive cross-sectional survey.

**Setting:**

All 12 regions in Morocco.

**Main outcome measures:**

Perceived priority drivers of mental health and well-being among youth.

**Results:**

A total of 1182 participants were included (mean age 20.5 years, 68.2% female, 85.7% from urban settings). Regarding health and nutrition, 46.3% valued sleep, 59.7% emphasised physical health, 53.1% highlighted access to quality healthcare and 56.5% prioritised clean air. In terms of connectedness and contribution, 75.7% rated family relationships as critical to their well-being, while 42.5% emphasised positive peer relationships. Regarding safety and supportive environments, 64.7% considered personal safety essential, 70% prioritised the fulfilment of basic needs and 63.7% valued personal information protection. For education and competence, 54.4% emphasised learning opportunities and 62.2% identified self-confidence as key drivers. Regarding agency and resilience, 59.4% valued independence, 68.5% stressed having a sense of purpose and 55% identified hope and optimism as key to their well-being. In digital well-being, 37.7% believed social media helped maintain connections, 38% viewed it as a learning tool while 31.6% reported it as a source of stress and anxiety

**Conclusions:**

This study provides valuable insights into priority drivers of youth mental health in Morocco from the perspective of Moroccan youth which should be the target for future interventions aiming to promote youth well-being. The findings contribute to the limited data on youth mental health in low and middle-income countries**,** highlighting the urgency for comprehensive mental health services and further research on subjective well-being.

**STRENGTHS AND LIMITATIONS OF THIS STUDY**

- This study provides valuable insights into priority drivers of youth mental health in Morocco which should be the target for future interventions aiming to promote youth well-being.
- The findings contribute to the limited data on youth mental health in low and middle-income countries, highlighting the urgency for comprehensive mental health services and further research.
- One of the limitations of the study is that the sample, while diverse geographically, has an overrepresentation of young people with higher education.
- Another limitation of the study relates to its digital survey reaching participants with access to mobile phones, with more limited participation from rural areas.
- The survey which was developed by the authors, based on the existing Adolescent Wellbeing Framework, was validated with a small sample prior to use as a measure.

## Background

 Mental health is defined as ‘a state of mental well-being that enables people to cope with the stresses of life, realise their abilities, learn well and work well, and contribute to their community’.^1^ Mental health means more than just the absence of problems but a process of systemic resilience, meaning that one is able to cope with change, continue to develop and thrive. Mental health struggles are a critical global issue, disproportionately affecting young people (15–25 years old) who bear a significant burden of these challenges because more than half of adult mental health disorders have their onset before or during adolescence.^1 2^ The consequences of these disorders can be severe, including self-harm and suicide, which is the third leading cause of mortality among youth globally.^2^ The drivers of mental health outcomes comprise a complex interplay of multiple neurological and psychosocial systems and social and environmental protective and risk factors, as well as available resources in mental health systems and their utilisation.^3^ Low- and middle-income countries (LMICs) face disproportionate gaps in their youth mental health systems and have environments with multiple risk factors and these landscapes of youth development create an additional challenge to addressing the unmet needs of young people.^4^

The drivers of mental health outcomes are identified as the interplay of protective and risk factors across the psychological, social, environmental, and biological systems in an individual’s life.^3^ These domains align with contemporary definitions of well-being that evaluate this subjective notion of 'well-being' across social, physical, cognitive, environmental, economic and psychological domains.^5^ Therefore, understanding a broader view of young people’s mental health and well-being requires understanding both their subjective evaluations of their own well-being and the drivers therein as well as the social and environmental determinants that shape their mental health outcomes. Relieving the burden of mental health conditions requires both treating young people and addressing the conditions that led to their mental health challenges in the first place.^6^

Young people (15–25 years old) in LMICs account for 90% of the world’s youth population.^7^ They often experience inequities in access to resources and opportunities and are more likely to face higher social and environmental risk factors.^4^ Existing research on mental health has explored drivers and social and environmental determinants linked to various mental health struggles. In a review of reviews conducted by Lund *et al*, social and environmental risk factors were organised in relation to the mental health outcomes they are related to. The environmental determinants included risk factors like economic inequity and its relation to low social status, worse physical health status and undernutrition and the relationship to mental disorder outcomes like depression, anxiety, substance use and psychosis. Social determinants included education, social cohesion, parenting and bullying and its relationship to depression, anxiety, psychosis and child and adolescent internalising disorders.^6^ However, limited research exists on the subjective evaluations of these drivers from the perspective of young people, centring their lived experiences and views of their own mental health.

The disparity in mental healthcare resources exacerbates the problem in LMIC contexts, with less than 2% of healthcare budgets being allocated to mental health.^1^ A critical issue is the shortage of mental health professionals in these regions, with an average of just one professional for every 200 000 people.^2^ In Morocco, the private sector, comprising 233 psychiatrists and only nine child psychiatrists,^8^ is largely concentrated in urban centres, making access to specialised care difficult for many.^8^ Non-governmental organisations and civil society organisations play a complementary role, with a few associations offering support for specific conditions like substance abuse, autism and disabilities.^9^ Mental health issues among Moroccan youth are further compounded by widespread substance abuse, depression, anxiety and other psychological disorders.

In Morocco, the Mediterranean Survey on Alcohol and Other Drug Use in Schools (MedSPAD)^9^ highlighted troubling rates of substance use, including tobacco (20% in boys, 6% in girls), cannabis (9.5% in boys) and psychotropic substances (4.4% in girls), starting as early as 15 years old.^9^ Moreover, the Global School-based Student Health Survey 2016^10^ revealed significant levels of depression, anxiety and insomnia, with 16.8% of students reporting sleep disturbances due to worry. Suicidal thoughts and behaviours are alarmingly prevalent, with 16% of urban students having seriously considered suicide.^10^

The current literature on what drives youth mental health and well-being in Morocco is limited and consists of small-scale studies largely focusing on accessible groups such as students.^11^,^14^ Despite efforts to prioritise youth mental health and well-being and increased interest in the area, the current evidence base is marked by significant gaps regarding the drivers of youth mental health and well-being. Thus, the aim of this study is to expand the existing evidence base by centering the perspectives of young people and to identify important drivers of mental health and well-being among Moroccan youth aged 18 to 24 across the 12 regions of Morocco. This study makes a significant contribution to youth mental health research in LMICs by providing large-scale data from a youth perspective compared with existing evidence and has important implications for research, programming and policy initiatives aimed at addressing the unique challenges faced by young people in these regions.

## Methods

### Study design and setting

A cross-sectional self-administered online survey was conducted among Moroccan young people between the ages of 18 and 24 years old across the 12 regions of Morocco (Tanger-Tétouan-Al Hoceïma, L'Oriental, Fès-Meknès, Rabat-Salé-Kénitra, Béni Mellal-Khénifra, Casablanca-Settat, Marrakech-Safi, Drâa-Tafilalet, Souss-Massa, Guelmim-Oued Noun, Laâyoune-Sakia El Hamra, Dakhla-Oued Ed-Dahab). The study period took place between November 2023 and February 2024. In order to ensure that the online survey would reach a wider distribution of young people and to ensure inclusion of respondents from all 12 regions.

Youth serving organisations are grassroots organisations local to different regions in Morocco. They operate within their communities through the regular organisation of community events, workshops and other youth-benefiting activities. As a result, they have an existing contact list of young people who receive announcements of these activities and had voluntarily signed up to be informed of the activities of the organisation. As most of these notices are shared through WhatsApp not an email newsletter as is more common in Global North settings, the youth serving organisations made contact with potential participants through WhatsApp. There was no sharing of confidential information directly through WhatsApp. WhatsApp as an accessible form of communication has double encryption which is why local grassroots organisations use it to connect with their communities. The electronic survey was created and shared through a secure institutional server with no third-party access to the raw data. The survey was analysed in a de-identified manner and did not collect the names of participants.

The online survey included two questions before the start of the questionnaire pertaining to consent. Participants indicated if they were between 18 and 24 years old and whether or not they consent to participating in the study. While the online survey was spread through a network of individuals through WhatsApp, the authors did not have any direct contact with participants.

### Study population

In this study, we included young people aged between 18 and 24 years old stratified by region from varying socio-demographic backgrounds residing in any of the 12 regions of Morocco, which strengthens geographic representativeness. The study used non-probabilistic, convenience sampling; however, in order to include young people representing the 12 regions of Morocco, sample size was allocated per regions and sub-regions, with sociodemographic characteristics such as place of residence, sex, education status being considered in participant outreach. This ensured a larger reach compared with existing studies while remaining within the feasibility and scope of the study.

### Conceptual framework

Well-being is an interdisciplinary construct that can be defined broadly, including both subjective evaluations of well-being and different indicators^15^ that fall under social and environmental determinants of well-being. Within the context of mental health, researchers evaluate well-being across social, physical, cognitive, environmental, economic and psychological domains.^5^ These domains, while mirroring social and environmental determinants of mental health outcomes, are self-evaluated subjectively by the individual, and thus fall under the definition of subjective well-being. The Adolescent Wellbeing Framework (AWF), developed in 2020 by the Partnership for Maternal, Newborn and Child Health, the WHO and the UN H6+partner agencies,^15^ defines adolescent well-being as: ‘Adolescents have the support, confidence, and resources to thrive in contexts of secure and healthy relationships, realising their full potential and rights’.

**Figure 1:**
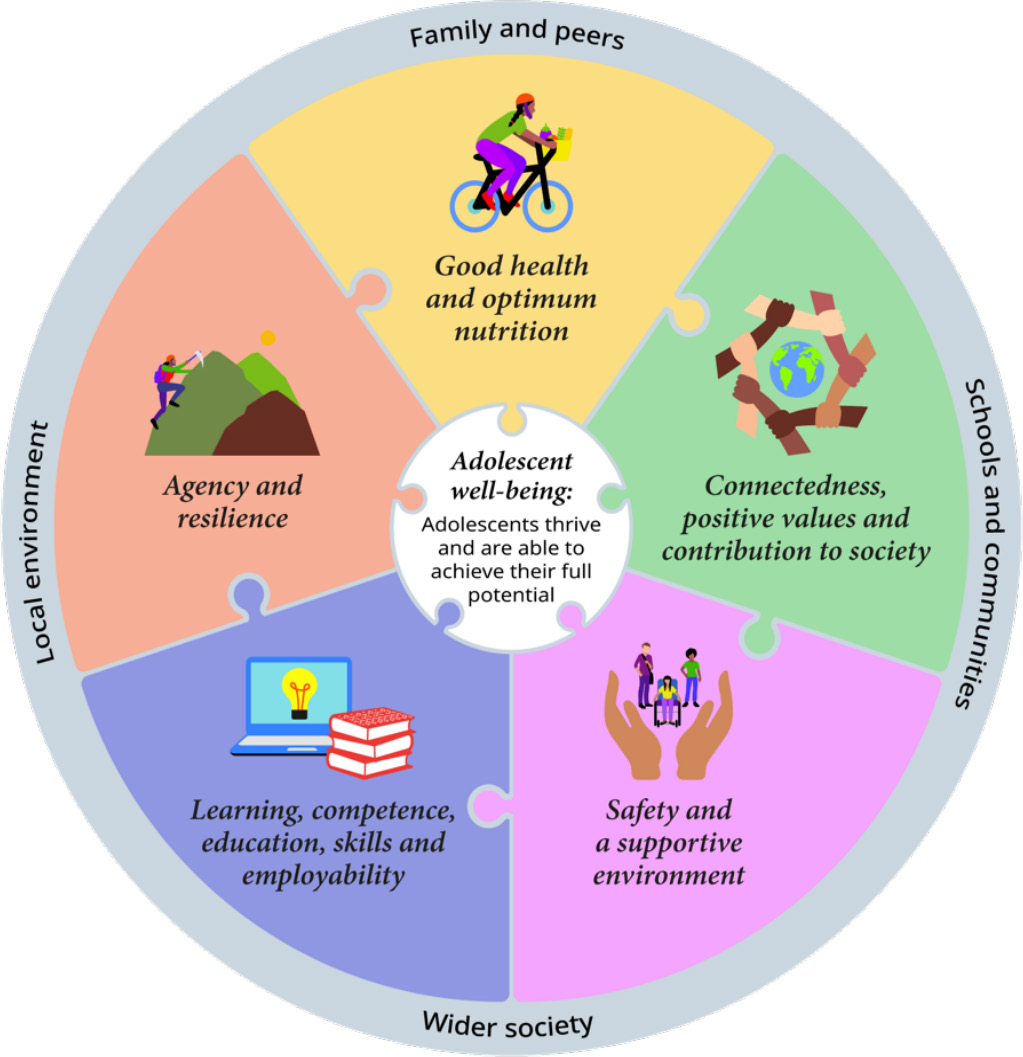
Adolescent Wellbeing Framework.^12^

The data collection survey used in this study was developed based on the AWF and subsequently validated with a group of 20 young Moroccans in a pilot study to check for reliability. The framework includes five components that determine adolescent well-being (figure 1): (1) good health and optimum nutrition; (2) connectedness, positive values and contribution to society; (3) safety and a supportive environment; (4) learning, competence, education, skills and employability; (5) agency (the capacity to take preferred action) and resilience (the ability to bounce back from adversity). While this is a relatively new framework, its selection for this study is based on its context relevance and its development in consultation with youth mental health experts, especially experts from LMIC settings and in consultation with young people. The original framework does not include assessment of young people’s digital life space; a sixth component, (6) digital well-being and literacy, was added to the survey, emergent from desk research on cyberbullying and consultations with Moroccan youth regarding their relationship with computers, devices and digital media, to enable an initial exploration of the determinants of well-being specific to the digital environment, virtual connectivity and internet use as a well-being driver. The term digital wellbeing, emerging from research on human-computer interaction, is an example of the interdisciplinary use of the term ‘wellbeing’ and is yet to have an agreed on operational definition.^16^

Consistent with established evidence that positive and negative dimensions of digital well-being represent distinct constructs, the Digital Well-being and Literacy domain was organised into two dimensions: Positive Digital Engagement and Self-Regulation (10 items measuring beneficial social media use, creative expression and intentional self-regulation behaviours) and Negative Digital Experiences (six items measuring social comparison, cyberbullying, problematic use and stress). The full survey has been added to online supplemental file 1).

To assess preliminary internal consistency reliability, Cronbach’s alpha coefficients and corrected item-total correlations were computed for each subscale among the 20 Moroccan young people who participated in the pilot validation phase. All five AWF subscales demonstrated excellent internal consistency: Good Health and Optimum Nutrition (α=0.938), Connectedness, Positive Values and Contribution to Society (α=0.952), Safety and Supportive Environment (α=0.956), Learning, Competence, Education and Employability (α=0.963) and Agency and Resilience (α=0.952). Corrected item-total correlations across these five subscales ranged from 0.558 to 0.956, with all items exceeding the conventional threshold of 0.30. For the Digital Well-being domain, both dimensions demonstrated acceptable to good internal consistency (Positive Digital Engagement and Self-Regulation: α=0.781; Negative Digital Experiences: α=0.802). All item-total correlations in the Negative Digital Experiences dimension exceeded the conventional threshold of 0.30 (range: 0.376–0.704). Three items in the Positive Digital Engagement dimension yielded correlations below 0.30, likely reflecting ceiling effects given the small pilot sample size rather than item misfit. Full item-level statistics are reported in online supplemental Tables S1–S6—online supplemental file 2).

### Data collection

12 young people were trained in participant recruitment and data collection, one for each region. These individuals were identified, selected and trained for their work within civil society and community organisations that have an established network within the target population. Within each of the 12 regions, data were collected using a self-administered online survey, using Microsoft Forms, which was made available in both Arabic and French. The survey was developed by the study team and validated by experts, based on the AWF (figure 1). For each item within the five domains (such as ‘Having an adult person in my life who I can trust’ under the Connectedness, positive values and contribution to society domain), with each domain containing between 6 and 16 items, participants indicated the level of importance the item rates in their well-being using a 5-point Likert scale from ‘extremely important’ to ‘not important at all’). The survey was piloted among 20 participants who met the inclusion criteria with feedback used to edit and modify the survey prior to its use for data collection and to ensure that well-being is understood in the context of mental health. The reproducibility and validity of the questionnaire have been assessed in this pilot study.

### Data analysis

Data collected from the online survey was collated into an excel spreadsheet and cleaned to ensure correct data entry. Although the survey’s network-style sharing makes the total youth reach difficult to determine, every participant who opted in answered all questions. Descriptive analysis was performed using the Jamovi 2.3.28 software and group comparisons using t-tests across age, sex and education level. Continuous variables were expressed in means and SD, and categorical variables were expressed in frequencies and percentages.

We conducted bivariate analyses using Pearson’s χ^2^ test to examine differences in well-being domains across socio-demographic characteristics (age, sex and level of education). In addition, we used ANOVA One-Way to examine any differences that exist between the regions across the six well-being domains. To facilitate this analysis, each well-being domain was transformed into a composite score based where by each item ranged from 0 (‘Not important at all’ or ‘Completely disagree’) to 4 (‘Extremely important’ or ‘Completely agree’). The well-being domains and their corresponding scores were as follows: good health and optimum nutrition (nine items; score range: 0–36), connectedness, positive values and contribution to society (nine items; score range: 0–36), safety and a supportive environment (seven items; score range: 0–28), learning, competence, education, skills and employability (eight items; score range: 0–32), agency and resilience (six items; score range: 0–24) and digital well-being (16 items; score range: 0–64).

### Patient and public involvement

Young people (18–25) were involved in validating the data collection tool, collecting data in their respective regions, and participating in a dissemination workshop.

## Results

### Socio-demographic characteristics

A total of 1182 (n=1182) responses were collected and analysed after data cleaning (table 1). The mean age of participants was 20.5 years old and the median was 20. A total of 68.2% of participants identified as female while only 31.8% were male. The majority of participants 85.7% were from an urban setting. 97.2% of participants reported their marital status as single. About 88.6% reported their highest education level being higher education or professional and /or vocational training with the majority of participants 81.7% being students at the time of the study.

**Table 1:** Sociodemographic characteristics of participants (n=1182)

Variables	Distribution N (%)
Age (years)	
18	236 (19.9)
19	198 (16.7)
20	225 (19.0)
21	152 (12.8)
22	107 (9.0)
23	130 (11.0)
24	135 (11.4)
Gender	
Female	807 (68.2)
Male	376 (31.8)
Place of residence	
Urban	1014 (85.7)
Rural	169 (14.3)
Marital status	
Single	1150 (97.2)
Married	27 (2.3)
Widowed	1 (0.1)
Divorced or separated	5 (0.4)
Level of education	
None	12 (1.0)
Primary	5 (0.4)
Secondary	26 (2.2)
High school	92 (7.8)
Higher education/professional training	1048 (88.6)
Professional status	
Student	967 (81.7)
Employed	105 (8.9)
Unemployed	12 (1.0)
Not in education, employment or training (NEET)	21 (1.8)
Searching for a job	78 (6.6)

### Mental health and well-being drivers

Online supplemental Table S7 in online supplemental file 2 presents how participants rated the importance of various drivers to their well-being. Below we present the top 10 results across all the components (Box 1).

Box 1 **What participants selected as key drivers of their mental health**

1. A good relationship with parents and family (n=896, 75.7%).
2. Access to essential needs such as food, water, a place to live, warmth, clothing (n=828, 70%).
3. A sense of purpose in life (n=810, 68.5%).
4. Feeling safe in daily life, whether at home, in the neighbourhood, online or at school or work (n=765, 64.7%).
5. Personal information is protected and is not shared without permission (n=754, 63.7%).
6. Having self-confidence and feeling capable to do things well (n=736, 62.2%).
7. Belief in the self and their ability to reach learning goals (n=708, 59.8%).
8. Physical health (n=706, 59.7%).
9. Not being exposed to violence (n=704, 59.5%).

In the first component, good health and optimum nutrition, 46.3% of participants rated sleeping at night to be extremely important to their well-being, 59.7% rated being physically healthy as extremely important, 53.1% identified having access to quality health services as extremely important and 56.5% reported breathing clean air as extremely important to their well-being.

In the second component, participants rated 10 items under connectedness, positive values and contribution to society. 75.7% identified having a relationship with parents and family as extremely important to their well-being and 42.5% rated having a positive relationship with peers and colleagues as very important.

In the third component, safety and a supportive environment, participants rated seven items under safety and a supportive environment and 64.7% identified feeling safe in daily life as extremely important to their well-being. 70% reported having essential needs such as food, water, a place to live, warmth, clothing, feeling safe and secure as extremely important to their well-being. 63.7% identified having their personal information protected as extremely important to their well-being.

In the fourth component, learning, competence, education, skills and employability, participants rated eight items under learning, competence, education, skills and employability. 54.4% rated getting to go to school and having chances to keep learning as extremely important to their well-being, 62.2% identified feeling self-confidence and feeling that they can do things well as extremely important to their well-being.

In the fifth component, agency and resilience, participants rated six items under agency and resilience. 59.4% rated feeling independent and capable of making their own decisions as extremely important to their well-being, 68.5% rated having a sense of purpose in their lives as extremely important to their well-being, and 55% rated having hope and optimism towards the future as extremely important.

Under the sixth component, digital well-being and literacy, participants rated 16 items using a scale from completely agree to strongly disagree. 37.7% of participants reported completely agreeing with social media helping them stay connected with friends and family and 48.2% agreed. 38% of participants rated social media as a useful tool for learning and accessing information as completely agree and 44.4% agreed. 43.4% selected completely agree and 33.6% agree in response to being cautious when sharing personal information on social media. 31.6% completely agreed and 32.6% agreed with social media being a source of stress and anxiety for them while 39% strongly disagreed with some people making fun of them on social media and affecting their well-being.

Table 2 summarises the mean scores of six mental health domains by age, sex and education level. No significant differences were found between age groups except for digital well-being, which was higher among youth under 20 (p=0.018). In contrast, significant sex differences were observed in all domains except digital well-being. Females consistently scored higher than males in learning competence, nutrition and health, agency, connectedness and safety (all p<0.001). Education level showed significant associations across all domains. Youth with higher education scored higher in all areas except digital well-being, which was unexpectedly higher among those with lower education (p=0.001). There were statistically significant differences between the regions across five out of the six well-being domains, with “Good health and optimum nutrition” being the exception (p=0.119)

**Table 2:** Comparison of well-being domains across key socio-demographic characteristics

Variable	Distribution mean (SD)									
	Age (years)			Sex			Education level			Region
	<20	>20	P value	Female	Male	P value	Higher education	Other	P value	P value
Learning, competence, education, skills and employability	26.25 (6.36)	25.98 (6.00)	0.474	26.84 (5.40)	24.43 (7.20)	<0.001	26.43 (5.43)	23.33 (9.64)	<0.001	0.029
Good health and optimum nutrition	27.40 (6.66)	27.61 (6.26)	0.606	28.15 (5.81)	26.21 (7.38)	<0.001	27.92 (5.75)	24.53 (9.70)	<0.001	0.119
Agency and resilience	20.39 (4.51)	20.52 (4.32)	0.624	20.97 (3.83)	19.41 (5.24)	<0.001	20.76 (3.87)	18.26 (6.87)	<0.001	<0.001
Connectedness, positive values and contribution to society	26.85 (7.27)	26.52 (6.77)	0.447	27.25 (6.40)	25.35 (7.87)	<0.001	26.86 (6.38)	24.93 (10.26)	0.034	<0.001
Safety and a supportive environment	23.48 (5.34)	23.14 (5.53)	0.3	24.32 (4.33)	21.00 (6.79)	<0.001	23.56 (4.97)	20.93 (8.00)	<0.001	0.003
Digital well-being and literacy	40.45 (10.63)	38.95 (10.36)	0.018	39.13 (9.76)	40.29 (11.85)	0.098	39.03 (9.96)	43.10 (13.35)	0.001	0.04

## Discussion

Data regarding the prevalence and social distribution of mental disorders, mental health, and well-being is not as well documented in LMICs, like Morocco, as it is in high-income countries.^17 18^ Understanding the drivers of mental health and well-being in specific contexts as well as the subjective evaluations of these drivers from the perspectives of key stakeholders and beneficiaries of the mental health system can inform the design of context-sensitive prevention strategies and population-level mental health initiatives. This study sought to fill the gap of research on social and environmental drivers that are associated with variations in well-being and mental health among young people, from the perspective of young people’s subjective evaluations of well-being. Surveying Moroccan youth between the ages of 18 and 24 years old across the 12 regions of Morocco, using the five components of the AWF and a sixth component on digital well-being, revealed the top ten drivers that Moroccan youth rated as highly important. The closer examination of the drivers given the highest rating of importance in ways that centre the lived experiences of young people allows comparison with global mental health research on drivers of mental health and the identification of any specificities that warrant further research for the Moroccan context

The results of this survey, under the first component of good health and optimum nutrition, and the second component of connectedness, positive values and contribution to society, show that young Moroccans rate very highly the importance of positive relationships within their families and with parents as well as access to essential needs such as food, water, a home, clothing and feeling safe. These results align with a systematic review of epidemiological studies that show a strong positive association between mental disorders and poverty in LMICs^19^ and studies showing that poorer mental health is found among populations who reported weak social support.^20^ A dominant hypothesis linking these mental health risk factors is that low socioeconomic status has been associated with greater exposure to frequent and prolonged stressors but these risk factors may be buffered with a good family relationship which is a source of social support.^21^ Therefore, for many participants in the study, who may be familiar with economic stressors, positive family and parental relationships rate higher as a priority slightly before basic needs. A study on the social determinants of mental health, conducted with youth in Indonesia, similarly highlights the importance of parental relationships with the young participants in the focus-group based study expressing that communication with parents and a sense of connection function as protective factors.^22^ These findings can provide evidence for programming to prioritise strengthening family relationships as a protective factor which is perceived by young people as contributing to their sense of resilience when facing adversity.

Under the third and fourth components, safety and a supportive environment and learning, competence, education, skills and employability, there were some interconnected findings. One of the determinants of poor mental health for young people is academic pressure and unsafe school environments^22^,^24^ which pose an added stressor for young people and may contribute to a cumulative burden of stress, which has been linked to poorer mental health outcomes. Participants in this study rated highly feeling a sense of purpose (meaning driving goal-directed behaviour), self-efficacy (the belief in one’s capacities) and capacity to learn (internal and external resources for knowledge and skill acquisition) as well as feeling safe and not being exposed to violence (at home from family or in school from teachers and peers or their neighbourhood through crime) as important to their mental health. Education plays an important role in a range of later life outcomes, including employment, income and community participation but lack of support in learning (such as the lack of additional study support outside the classroom) or feeling unsafe in school due to bullying may reflect a reciprocal association between academic challenges and reduced confidence and self-efficacy.^2325^,^28^ Literature from high-income countries^29^ as well as from other LMICs^30^ showcase a link between academic pressure and the way it is associated with mental health challenges among young people, sense of purpose in life, confidence and self-efficacy which have been correlated with adverse mental health outcomes.

Connecting the fifth component and sixth component, agency (the ability to take preferred action) and resilience (the ability to bounce back from adversity) as well as digital well-being and literacy, young people rated privacy of personal information as highly important to them. Due to its implications for their ability to have agency and make their own life choices, this area of digital well-being requires further research to understand the relationship between perceived digital privacy concerns and young people reported mental health and well-being in LMICs like Morocco.

The findings of this study reflect the subjective experiences of young people and their tacit awareness of the protective factors that help mitigate or serve as protective factors in the presence of known risks and social determinants in their environment. They rate supportive family relationships as mattering more to their mental health and well-being than not being exposed to poverty and a sense of purpose and capacity to learn and do more highly than safety in schools. This is an example of young people rating protective factors that may buffer risk factors in their social and environmental systems as more important for their subjective well-being than the reduction of the risk factors. While all the drivers together paint a picture of what young people need to promote their mental health and positive development and the differences between the highly rated items may not be large enough for statistical significance, the results of this study can inform the priorities of intervention and programming in Morocco. It highlights the need to strengthen the protective factors that matter to young people and are perceived by participants as playing a meaningful role in shaping their mental health and sense of resilience, especially when they may be exposed to stressors like poverty and unsafe school environments.

The study’s findings must be considered in light of several limitations. The participants were recruited through non-probabilistic, convenience sampling and in order to include young people representing the 12 regions of Morocco sample size was allocated per regions and sub-regions, with specified sociodemographic characteristics. Therefore, there was a skew towards young people with access to digital devices who could be reached through this sampling method, thus underrepresenting rural participants. The data collection methodology was a digital survey, rather than an in-person data collection method, which resulted in an overrepresentation of participants with higher education. As outreach to potential participants was done through youth serving organisations who sent out the electronic survey to their local community of young people who participate in the organisation’s activities, the gender of study participants skewed to primarily female participants as they tend to be overrepresented in activity participation. Therefore, this study, while reaching a larger number of participants than existing studies, is not representative of all Moroccan youth with differing abilities, needs, or socio-economic backgrounds, thus limiting the national generalisability of the study’s results. Only 1.8% of respondents identified as NEET, indicating that the perspectives of this group may be underrepresented. Additionally, with only 14.3% of participants coming from rural contexts, the study may not fully capture the unique challenges and experiences of rural youth, who often face different socio-economic and infrastructural conditions compared with their urban counterparts. The survey used in this study was developed by the research team based on the AWF which is a relatively new framework that is yet in the process of developing validated measures. The survey developed for this study was validated with a small sample of n=20. As such, the lack of robustly psychometrically validated surveys introduces potential concerns regarding the capacity to compare the internal and external validity of the findings. The lack of other studies published thus far using this framework limits the capacity to ascertain the criterion validity of the survey.

Nevertheless, this study is the first of its kind in Morocco. It is large-scale with data collection reaching all 12 regions in Morocco rather than remaining limited to large urban centres. The study centres the perspectives of young people and their subjective evaluations of the drivers they consider to be a priority to their own well-being. It can provide important guidance for further research to better understand drivers of well-being, policy regarding protections for youth well-being and practice for intervention and programme design to promote well-being. Data on well-being is not systematically collected in Morocco therefore it is likely to be fragmented and focused on the prevalence of psychiatric disorders. However, the Ministry of Health in Morocco is developing a multi-sectoral strategy and this study is in line with the current efforts to inform youth related aspects of this strategy. This study can help inform the training of counsellors and mental health professionals^31^ by providing a list of priority areas for psychoeducation and prevention programmes in schools and community settings. It can also expand the conversation in Morocco beyond mental illnesses to focus reforms on addressing social and environmental determinants of mental health and well-being with local guidance on drivers of mental health to help address inequities which impact young people’s mental health. It can be used to guide youth programming, especially in providing context-relevant interventions that support the development of positive relationships with parents and family members such as in the PTP programme, that build a sense of purpose, self-efficacy and capacity.^32^ It can also inform policy that seeks to address inequities through the reduction of poverty and creating safer environments for young people in schools, their communities and at home by opening new avenues of institutional collaboration through the lens of youth mental health. Having the perspectives of Moroccan youth from across 12 regions speaking to the drivers of mental health from their perspective can help enhance the inclusion of youth lived experiences (young people’s subjective evaluations and expressions of their experiences and perspectives) in policy and practice and encourage further participatory research.

## Conclusions

This cross-sectional study on the perceived priority drivers of youth mental health and well-being addressed a key gap in literature by identifying important drivers of mental health and well-being from the perspective and lived experience of Moroccan youth between the ages of 18 and 24 years old across the 12 regions of Morocco. It is one of the first to use the AWF in data collection in an LMIC setting and expand on the framework’s five components with a sixth addressing digital well-being. The findings of this study included insightful perspectives from young people in Morocco on how they rate the drivers that matter most to them, which include the importance of family and parental relationships, having a sense of purpose, feeling safe at home, in their neighbourhoods and in school and the protection of personal information online. These findings suggest that young people recognise the influence of social and environmental determinants on their mental health but they also value protective factors that may play a supportive role in how they navigate social and environmental risks and adversity with resilience which opens avenues for further research in the Moroccan context. The contribution of this study is significant in that it contributes to filling a large gap of data in the area of youth mental health in Morocco and in LMICs and points to priority areas for both research, intervention and policy development.

## Supplementary material

10.1136/bmjopen-2025-110683 online supplemental file 1

10.1136/bmjopen-2025-110683 online supplemental file 2

## Data Availability

Data are available upon reasonable request.
