# Supplementary material for: Drivers of youth mental health and wellbeing: a large-scale cross-sectional study in Morocco
Source: BMJ Open. 2026 Jun 9;16(6):e110683. doi: 10.1136/bmjopen-2025-110683 (PMC13264952; doi:10.1136/bmjopen-2025-110683)
Supplement: online supplemental file 1 [file bmjopen-16-6-s001.pdf]

Étude sur la santé mentale et le bien-être des jeunes au Maroc

14 déc. 2023

Cette étude est menée par l'Université Mohammed VI des Sciences et de la Santé et le Centre Mo
pour la Recherche et l'Innovation. Vos données seront traitées de manière complètement confide
vous acceptez de participer, vous allez répondre à une série de questions. Cela prendra environ 2

* Obligatoire

1. Votre âge est entre 18 et 24 ans

- ◯ Oui
- ◯ Non

2. Consentement *

◯ J'accepte de participer à l'enquête.

Informations générales

3. Votre âge *

- 18
- 19
- 20
- 21
- 22
- 23
- 24

4. Sexe *

- Homme
- Femme

5. Lieu de résidence *

- Urbain
- Rural

6. Ville de résidence *

7. Numéro de téléphone *

8. Vous êtes : *

- Célibataire
- Marié(e)
- Veuf (veuve)
- Divorcé(e) ou séparé(e)

9. Quel est le plus haut niveau d'éducation que vous avez atteint ? *

- Non-scolarisé(e)
- Primaire
- Secondaire
- Lycée
- Enseignement supérieur (Université) / Formation professionnelle

10. Actuellement, vous êtes: *

- ◯ Étudiant(e)
- ◯ Employé(e)
- ◯ Sans emploi
- ◯ Ni en éducation, ni en emploi, ni en formation

Déterminants de santé mentale et de bien-être

11. Dans quelle mesure les éléments suivants sont-ils importants pour votre bien-être ? *

	Très important	Important	Modérément important	Légèrement important	Pas important
**Avoir une alimentation variée et équilibrée**	○	○	○	○	○
**Bien dormir la nuit**	○	○	○	○	○
**Faire du sport**	○	○	○	○	○
**Être satisfait(e) de mon apparence**	○	○	○	○	○
**Être en bonne santé physique**	○	○	○	○	○
**Avoir accès à des informations sur la santé**	○	○	○	○	○
**Avoir accès à des services de santé de qualité**	○	○	○	○	○
**Avoir accès aux espaces verts**	○	○	○	○	○
**Respirer de l'air pur**	○	○	○	○	○

12. **Dans quelle mesure les éléments suivants sont-ils importants pour votre bien-être ?** *

	Très important	Important	Modérément important	Légèrement important	Pas important
**Avoir de bonnes relations avec mes parents et ma famille**	◯	◯	◯	◯	◯
**Avoir une bonne relation avec mes enseignants/ professeurs/ employeur**	◯	◯	◯	◯	◯
**Avoir une bonne relation avec mes pairs/collègues**	◯	◯	◯	◯	◯
**Se sentir intégré(e) dans ma communauté, mon école/université ou mon milieu de travail**	◯	◯	◯	◯	◯
**Avoir un adulte dans ma vie en qui je peux avoir confiance**	◯	◯	◯	◯	◯
**Se sentir accepté(e), respecté(e) et valorisé(e) par les autres**	◯	◯	◯	◯	◯
**Participer à des activités sociales et [illegible]**					

culturelles dans la communauté, à l'école/université ou au travail

Avoir l'opportunité de participer à la prise de décision et que mes idées soient valorisées et respectées

Avoir la chance d'apprendre l'empathie, de se faire des amis et de devenir plus compréhensif/ve

13. **Dans quelle mesure les éléments suivants sont-ils importants pour votre bien-être ?** *

	Très important	Important	Modérément important	Légèrement important	Pas important
**Ne pas être exposé(e) à la violence (y compris l'intimidation, le harcèlement en ligne, les abus physiques, sexuels, verbaux et la violence émotionnelle)**	○	○	○	○	○
**Me sentir en sécurité dans ma vie quotidienne, que ce soit à la maison, dans mon quartier, en ligne, à l'école ou au travail.**	○	○	○	○	○
**Être traité comme les autres et sans discrimination**	○	○	○	○	○
**Se sentir en sécurité pour m'exprimer et être moi-même**	○	○	○	○	○
**Avoir les besoins nécessaires comme de la nourriture, de l'eau, un endroit où vivre, de la chaleur, des**	○	○	○	○	○

**vêtements et me sentir en sécurité**					
**Mes informations personnelles sont protégées et ne sont pas partagées sans mon autorisation**	○	○	○	○	○
**Avoir accès à des activités de loisirs et à des opportunités de développement personnel**	○	○	○	○	○

14. **Dans quelle mesure les éléments suivants sont-ils importants pour votre bien-être ?** *

	Très important	Important	Modérément important	Légèrement important	Pas important
**Pouvoir aller à l'école et avoir des opportunités pour continuer d'apprendre même après, que ce soit en classe ou par d'autres moyens d'apprentissage**	○	○	○	○	○
**Bénéficier de l'accompagnement pour rester motivé(e) et continuer à apprendre**	○	○	○	○	○
**Avoir la possibilité de développer les outils et les compétences nécessaires pour réussir**	○	○	○	○	○
**Avoir confiance en soi et se sentir capable de bien faire les choses**	○	○	○	○	○
**Acquérir des compétences pratiques pour le travail**	○	○	○	○	○
**Travailler dans des emplois et**	○	○	○	○	○

**des entreprises adaptés à mon âge**	◯	◯	◯	◯	◯
**Être satisfait à l'égard de mon apprentissage et de mes compétences**	◯	◯	◯	◯	◯
**Croire en moi et en ma capacité à atteindre mes objectifs d'apprentissage**	◯	◯	◯	◯	◯

15. **Dans quelle mesure les éléments suivants sont-ils importants pour votre bien-être ?** *

	Très important	Important	Modérément important	Légèrement important	Pas important
**Se sentir indépendant et capable de prendre mes propre décisions**	○	○	○	○	○
**Sentir que j'ai le pouvoir d'accomplir des choses et avoir confiance en moi (que ce soit avec des amis, la famille, ou dans la prise des décisions)**	○	○	○	○	○
**Avoir de l'espoir et de l'optimisme à l'égard de l'avenir**	○	○	○	○	○
**Avoir un but dans ma vie**	○	○	○	○	○
**Avoir des opportunités de développer la capacité à relever les défis de la vie actuelle et future**	○	○	○	○	○
**Avoir des chances d'atteindre mon plein potentiel maintenant et dans le future**	○	○	○	○	○

16. **A quel point vous êtes d’accord ou pas d’accord avec les propositions suivantes ?** *

	Tout à fait d'accord	D'accord	Indécis(e)	Pas d'accord	Pas du tout d'accord
Les réseaux sociaux m'aident à rester en contact avec mes amis et ma famille	○	○	○	○	○
Je ressens la pression de montrer une vie parfaite sur les réseaux sociaux	○	○	○	○	○
Les réseaux sociaux ont un impact positif sur mon estime de soi	○	○	○	○	○
Certaines personnes se moquent de moi sur les réseaux sociaux, ce qui affecte mon bien-être	○	○	○	○	○
J'ai du mal à me déconnecter des réseaux sociaux, même lorsque j'en ai envie	○	○	○	○	○
Les réseaux sociaux me permettent d’exprimer ma créativité et mes centres d’intérêts	○	○	○	○	○

**Me comparer aux autres sur les réseaux sociaux me fait sentir inadéquat(e)**	◯	◯	◯	◯	◯
**Les réseaux sociaux sont un outil utile pour apprendre et accéder à l’information**	◯	◯	◯	◯	◯
**Je suis prudent concernant le partage de mes informations personnelles sur les réseaux sociaux**	◯	◯	◯	◯	◯
**Je reçois du soutien et des encouragements de communautés ou de groupes en ligne**	◯	◯	◯	◯	◯
**Je me sens souvent submergé(e) par le flux constant d’informations sur les réseaux sociaux**	◯	◯	◯	◯	◯
**Les réseaux sociaux m'aident à découvrir de nouveaux loisirs et intérêts.**	◯	◯	◯	◯	◯
**J'ai pris des pauses sur**					

**les réseaux sociaux pour améliorer mon bien-être**	○	○	○	○	○
**Les réseaux sociaux peuvent être pour moi une source de stress et d'anxiété**	○	○	○	○	○
**Je suis conscient de mon temps d'écran et j'essaie de l'équilibrer avec d'autres activités**	○	○	○	○	○
**J'ai utilisé les réseaux sociaux pour demander des conseils ou des orientations sur des problèmes personnels**	○	○	○	○	○

Connaissances en santé mentale et sources d'information utilisées

17. **A votre avis:**

	Vrai	Faux	Je ne sais pas
**La santé mentale est une composante de la santé et la maladie mentale est comme toute autre maladie**	○	○	○
**Les pensées suicidaires ou les tentatives de suicide sont l'un des problèmes psychologiques**	○	○	○
**Les problèmes psychologiques ou la maladie mentale peuvent apparaître très tôt**	○	○	○
**À cause du harcèlement ou des abus, on peut développer des problèmes psychologiques et des troubles mentaux**	○	○	○
**Les patients atteints de troubles mentaux ne sont pas toujours tristes**	○	○	○
**Une [illegible]**			

personne déprimée se [illegible]

18. **À quelle fréquence recherchez-vous des informations sur la santé mentale?** *

- ◯ Toujours
- ◯ Très souvent
- ◯ Parfois
- ◯ Rarement
- ◯ Jamais

19. **Quelles sources d’information utilisez-vous pour obtenir des informations sur la santé mentale ?** *

- [ ] Conseiller(s) scolaire(s) ou universitaire(s) ou dans le milieu du travail
- [ ] Professionnel de la santé mentale (ex : médecin, psychologue)
- [ ] Organisations de santé mentale
- [ ] Enseignants et professeurs
- [ ] Réseaux sociaux
- [ ] Applications de santé mentale
- [ ] Articles en ligne
- [ ] Télévision ou radio
- [ ] Livres ou revues
- [ ] Podcasts
- [ ] Coachs de développement personnel
- [ ] Lignes d'assistance ou lignes vertes
- [ ] Adultes en lesquelles vous faites confiance
- [ ] Ami(e)s et famille
- [ ] Autre (veuillez préciser)...

20. Autre (veuillez préciser):

Engagement des jeunes

21. **À quelle fréquence participez-vous à des activités relative la santé mentale et le bien-être ?** *

- ◯ Toujours
- ◯ Très souvent
- ◯ Parfois
- ◯ Rarement
- ◯ Jamais

22. **Dans quelle mesure cela s'applique-t-il à vous** *

	Toujours	Très souvent	Parfois	Rarement	Jamais
**Je participe à la conception des programmes et des activités liés santé mentale**	○	○	○	○	○
**Je participe à la mise en œuvre de programmes de santé mentale**	○	○	○	○	○
**Je participe au suivi et à l'évaluation des programmes de santé mentale**	○	○	○	○	○
**Je participe à activement la sensibilisatio n autour de la santé mentale**	○	○	○	○	○
**Je fais partie des groupes qui prennent des décisions concernant la santé mentale**	○	○	○	○	○

23. **Dans quelle mesure êtes-vous d'accord avec ce qui suit :** *

	Tout à fait d'accord	D'accord	Indécis(e)	Pas d'accord	Pas du tout d'accord
Je pourrais être jugé par les autres si je parle de ma santé mentale	○	○	○	○	○
Je ne sais pas toujours où trouver de l'aide lorsque j'en ai besoin	○	○	○	○	○
Je crois qu'il n'y a pas assez de ressources et d'investissement dans les programmes de santé mentale	○	○	○	○	○
Parfois, je ne peux pas me rendre dans les endroits où un soutien en santé mentale est disponible parce que c'est trop loin ou parce que cela coûte trop cher	○	○	○	○	○
Je pourrais avoir du mal à trouver de l'aide si les gens autour de moi parlent des langues différentes ou ont des origines [illegible]	○	○	○	○	○

**différentes**					
**Je ne comprends pas ce que signifie réellement la santé mentale ni pourquoi elle est importante**	○	○	○	○	○
**Je crains que si je demande de l'aide ou si j'essaie d'aider les autres, je puisse être traité injustement ou jugé par les autres**	○	○	○	○	○
**J'ai un emploi du temps chargé avec l'école/l'université/le travail, donc je n'ai pas le temps pour m'impliquer dans des activités de santé mentale**	○	○	○	○	○
**J'ai peut-être l'impression d'être la seule qui s'intéresse la santé mentale**	○	○	○	○	○
**J'ai déjà eu une mauvaise expérience avec les services de santé mentale, ce qui me fait hésiter à réessayer**	○	○	○	○	○

Intérêts, préparation et volonté des jeunes à agir et à collaborer

24. **Quel est votre niveau d’intérêt à agir pour améliorer la santé mentale au Maroc ?** *

1	2	3	4	5	6	7	8	9	10

25. **A votre avis, quel est le niveau d'importance pour les jeunes de plaider en faveur de l'amélioration de la santé mentale des jeunes au Maroc?** *

1	2	3	4	5	6	7	8	9	10

26. **Dans quelle mesure êtes-vous prêt à participer à des activités et initiatives visant à améliorer la santé mentale des jeunes au Maroc ?** *

1	2	3	4	5	6	7	8	9	10

27. **Selon vous, quels obstacles empêchent les jeunes de parler ouvertement de santé mentale ?** *

- [ ] Manque de temps
- [ ] Stigmatisation et peur du jugement
- [ ] Manque de connaissances ou de compréhension des problèmes de santé mentale
- [ ] Manque d'accès aux services de santé mentale
- [ ] Peur de perdre des relations ou des opportunités
- [ ] Manque de soutien de la part des amis et de la famille
- [ ] Incapacité à trouver les mots justes pour s'exprimer
- [ ] L'impression que ça ne sert à rien d'en parler
- [ ] Avoir l'impression que c'est trop personnel ou privé pour en parler
- [ ] L'impression que ce n'est pas un "vrai" problème
- [ ] Autre (veuillez préciser)...

28. Autre (veuillez préciser):

29. **De quel soutien auriez-vous besoin pour participer à des initiatives ou des activités relatives à la santé mentale des jeunes au Maroc?** *

- [ ] Accès à des professionnels ou à des conseillers en santé mentale
- [ ] Soutien des amis et de la famille
- [ ] Information et éducation sur les problèmes de santé mentale
- [ ] Un espace sécurisé pour parler des problèmes de santé mentale
- [ ] Aide financière pour les services de santé mentale
- [ ] Changements de politiques pour améliorer l’accès aux services de santé mentale
- [ ] Autre (veuillez préciser)

30. Autre (veuillez préciser):

31. **Dans quelle mesure seriez-vous prêt à collaborer avec d'autres parties prenantes, telles que le gouvernement, la société civile ou les entreprises, pour améliorer la santé mentale des jeunes au Maroc ?**
*

	Extrêmement prêt	Très prêt	Modérément prêt	Légèrement prêt	Pas du tout prêt
Ministères et autres agences gouvernementales	○	○	○	○	○
Prestataires de services de santé mentale, tels que les hôpitaux, les cliniques et les centres d'écoute	○	○	○	○	○
Organisations et associations de la société civile	○	○	○	○	○
Chercheurs et universitaires en santé mentale	○	○	○	○	○
Acteurs dans les médias et la communication	○	○	○	○	○
Entreprises privées	○	○	○	○	○
Personnes et organisations religieuses	○	○	○	○	○
Étudiants et groupes de jeunes	○	○	○	○	○

Ce contenu n’a pas été créé ni n’est approuvé par Microsoft. Les données que vous soumettez sont envoyées au propriétaire du formulaire.

Microsoft Forms
