# Supplementary material for: Drivers of youth mental health and wellbeing: a large-scale cross-sectional study in Morocco
Source: BMJ Open. 2026 Jun 9;16(6):e110683. doi: 10.1136/bmjopen-2025-110683 (PMC13264952; doi:10.1136/bmjopen-2025-110683)
Supplement: online supplemental file 2 [file bmjopen-16-6-s002.docx]

**Supplementary File 1**

Table of Contents

[Reliability analysis 1](#_Toc230166539)

[Supplementary results table 8](#_Toc230166540)

# **Reliability analysis**

- N items = 9
- Mean inter-item r = 0.648
- Cronbach's α = 0.938

***Table S1a. Corrected Item-Total Correlations***

| **Item** | **Corrected Item-Total r** |
| --- | --- |
| **Balanced diet** | 0.558 |
| **Sleeping well** | 0.565 |
| **Sport and exercise** | 0.871 |
| **Satisfied with appearance** | 0.871 |
| **Physically healthy** | 0.759 |
| **Access to health info** | 0.763 |
| **Access to health services** | 0.907 |
| **Access to green spaces** | 0.934 |
| **Clean air** | 0.775 |

*Note. r = corrected item-total correlation*

***Table S1b. Inter-Item Correlation Matrix***

|  | **1** | **2** | **3** | **4** | **5** | **6** | **7** | **8** | **9** |
| --- | --- | --- | --- | --- | --- | --- | --- | --- | --- |
| **1. Balanced diet** | — | 0.250 | 0.652 | 0.412 | 0.233 | 0.635 | 0.672 | 0.521 | 0.368 |
| **2. Sleeping well** | 0.250 | — | 0.436 | 0.711 | 0.755 | 0.257 | 0.434 | 0.539 | 0.610 |
| **3. Sport and exercise** | 0.652 | 0.436 | — | 0.865 | 0.556 | 0.826 | 0.885 | 0.827 | 0.599 |
| **4. Satisfied with appearance** | 0.412 | 0.711 | 0.865 | — | 0.755 | 0.665 | 0.824 | 0.865 | 0.666 |
| **5. Physically healthy** | 0.233 | 0.755 | 0.556 | 0.755 | — | 0.469 | 0.640 | 0.843 | 0.944 |
| **6. Access to health info** | 0.635 | 0.257 | 0.826 | 0.665 | 0.469 | — | 0.857 | 0.772 | 0.550 |
| **7. Access to health services** | 0.672 | 0.434 | 0.885 | 0.824 | 0.640 | 0.857 | — | 0.896 | 0.660 |
| **8. Access to green spaces** | 0.521 | 0.539 | 0.827 | 0.865 | 0.843 | 0.772 | 0.896 | — | 0.864 |
| **9. Clean air** | 0.368 | 0.610 | 0.599 | 0.666 | 0.944 | 0.550 | 0.660 | 0.864 | — |

*Pearson correlations between individual items. N = 20.*

**Subscale 2: Connectedness, Positive Values and Contribution to Society**

- N items = 9
- Cronbach's α = 0.952
- Mean inter-item r = 0.691

***Table S2a. Corrected Item-Total Correlations***

| **Item** | **Corrected Item-Total r** |
| --- | --- |
| **Relations w/ parents/family** | 0.847 |
| **Relations w/ teachers/employer** | 0.852 |
| **Relations w/ peers** | 0.849 |
| **Sense of belonging** | 0.779 |
| **Trusted adult in life** | 0.825 |
| **Accepted and respected by others** | 0.863 |
| **Social/cultural participation** | 0.668 |
| **Participation in decision-making** | 0.768 |
| **Learning empathy and friendship** | 0.832 |

***Table S2b. Inter-Item Correlation Matrix***

|  | **1** | **2** | **3** | **4** | **5** | **6** | **7** | **8** | **9** |
| --- | --- | --- | --- | --- | --- | --- | --- | --- | --- |
| **1. Relations w/ parents/family** | — | 0.781 | 0.773 | 0.714 | 0.711 | 0.709 | 0.653 | 0.668 | 0.741 |
| **2. Relations w/ teachers/employer** | 0.781 | — | 0.785 | 0.906 | 0.680 | 0.796 | 0.446 | 0.567 | 0.789 |
| **3. Relations w/ peers** | 0.773 | 0.785 | — | 0.764 | 0.701 | 0.848 | 0.579 | 0.651 | 0.659 |
| **4. Sense of belonging** | 0.714 | 0.906 | 0.764 | — | 0.647 | 0.783 | 0.329 | 0.513 | 0.648 |
| **5. Trusted adult in life** | 0.711 | 0.680 | 0.701 | 0.647 | — | 0.770 | 0.688 | 0.675 | 0.763 |
| **6. Accepted and respected by others** | 0.709 | 0.796 | 0.848 | 0.783 | 0.770 | — | 0.531 | 0.690 | 0.710 |
| **7. Social/cultural participation** | 0.653 | 0.446 | 0.579 | 0.329 | 0.688 | 0.531 | — | 0.841 | 0.660 |
| **8. Participation in decision-making** | 0.668 | 0.567 | 0.651 | 0.513 | 0.675 | 0.690 | 0.841 | — | 0.703 |
| **9. Learning empathy and friendship** | 0.741 | 0.789 | 0.659 | 0.648 | 0.763 | 0.710 | 0.660 | 0.703 | — |

**Subscale 3: Safety and Supportive Environment**

- N items = 7
- Cronbach's α = 0.956
- Mean inter-item r = 0.764

***Table S3a. Corrected Item-Total Correlations***

| **Item** | **Corrected Item-Total r** |
| --- | --- |
| **Not exposed to violence** | 0.774 |
| **Feeling safe daily** | 0.956 |
| **Treated without discrimination** | 0.833 |
| **Safe to express myself** | 0.940 |
| **Basic needs met** | 0.879 |
| **Personal info protected** | 0.782 |
| **Access to leisure and development** | 0.800 |

***Table S3b. Inter-Item Correlation Matrix***

|  | **1** | **2** | **3** | **4** | **5** | **6** | **7** |
| --- | --- | --- | --- | --- | --- | --- | --- |
| **1. Not exposed to violence** | — | 0.859 | 0.724 | 0.717 | 0.693 | 0.649 | 0.581 |
| **2. Feeling safe daily** | 0.859 | — | 0.843 | 0.890 | 0.924 | 0.755 | 0.787 |
| **3. Treated without discrimination** | 0.724 | 0.843 | — | 0.849 | 0.813 | 0.640 | 0.635 |
| **4. Safe to express myself** | 0.717 | 0.890 | 0.849 | — | 0.874 | 0.808 | 0.849 |
| **5. Basic needs met** | 0.693 | 0.924 | 0.813 | 0.874 | — | 0.665 | 0.757 |
| **6. Personal info protected** | 0.649 | 0.755 | 0.640 | 0.808 | 0.665 | — | 0.733 |
| **7. Access to leisure and development** | 0.581 | 0.787 | 0.635 | 0.849 | 0.757 | 0.733 | — |

**Subscale 4: Learning, Competence, Education and Employability**

- N items = 8
- Cronbach's α = 0.963
- Mean inter-item r = 0.770

***Table S4a. Corrected Item-Total Correlations***

| **Item** | **Corrected Item-Total r** |
| --- | --- |
| **Access to schooling/learning** | 0.898 |
| **Support to stay motivated** | 0.772 |
| **Tools and skills to succeed** | 0.789 |
| **Self-confidence** | 0.858 |
| **Practical work skills** | 0.877 |
| **Age-appropriate work** | 0.859 |
| **Satisfied with learning** | 0.909 |
| **Belief in learning goals** | 0.912 |

***Table S4b. Inter-Item Correlation Matrix***

|  | **1** | **2** | **3** | **4** | **5** | **6** | **7** | **8** |
| --- | --- | --- | --- | --- | --- | --- | --- | --- |
| **1. Access to schooling/learning** | — | 0.785 | 0.709 | 0.821 | 0.824 | 0.818 | 0.836 | 0.806 |
| **2. Support to stay motivated** | 0.785 | — | 0.628 | 0.732 | 0.628 | 0.674 | 0.739 | 0.716 |
| **3. Tools and skills to succeed** | 0.709 | 0.628 | — | 0.766 | 0.670 | 0.677 | 0.793 | 0.757 |
| **4. Self-confidence** | 0.821 | 0.732 | 0.766 | — | 0.766 | 0.710 | 0.727 | 0.865 |
| **5. Practical work skills** | 0.824 | 0.628 | 0.670 | 0.766 | — | 0.884 | 0.845 | 0.871 |
| **6. Age-appropriate work** | 0.818 | 0.674 | 0.677 | 0.710 | 0.884 | — | 0.839 | 0.785 |
| **7. Satisfied with learning** | 0.836 | 0.739 | 0.793 | 0.727 | 0.845 | 0.839 | — | 0.881 |
| **8. Belief in learning goals** | 0.806 | 0.716 | 0.757 | 0.865 | 0.871 | 0.785 | 0.881 | — |

**Subscale 5: Agency and Resilience**

- N items = 6
- Cronbach's α = 0.952
- Mean inter-item r = 0.777

***Table S5a. Corrected Item-Total Correlations***

| **Item** | **Corrected Item-Total r** |
| --- | --- |
| **Feeling independent** | 0.857 |
| **Feeling empowered** | 0.743 |
| **Hope and optimism** | 0.804 |
| **Sense of purpose** | 0.925 |
| **Handle life challenges** | 0.904 |
| **Reach full potential** | 0.905 |

***Table S5b. Inter-Item Correlation Matrix***

|  | **1** | **2** | **3** | **4** | **5** | **6** |
| --- | --- | --- | --- | --- | --- | --- |
| **1. Feeling independent** | — | 0.741 | 0.662 | 0.828 | 0.795 | 0.864 |
| **2. Feeling empowered** | 0.741 | — | 0.591 | 0.709 | 0.708 | 0.696 |
| **3. Hope and optimism** | 0.662 | 0.591 | — | 0.832 | 0.846 | 0.760 |
| **4. Sense of purpose** | 0.828 | 0.709 | 0.832 | — | 0.865 | 0.912 |
| **5. Handle life challenges** | 0.795 | 0.708 | 0.846 | 0.865 | — | 0.845 |
| **6. Reach full potential** | 0.864 | 0.696 | 0.760 | 0.912 | 0.845 | — |

**Subscale 6: Digital Well-being and Literacy**

***Table S6a. Corrected Item-Total Correlations***

| **Item** | **Corrected Item-Total r** |
| --- | --- |
| **Dimension 1: Positive Digital Engagement & Self-Regulation** (N = 10, α = 0.781, Mean inter-item r = 0.253) | |
| **1. Social media helps me stay connected with my friends and family** | 0.170* |
| **2. Social media has a positive impact on my self-esteem** | 0.440 |
| **3. Social media provides a platform for me to express my creativity and interests** | 0.624 |
| **4. Social media is a useful tool for learning and accessing information** | 0.113* |
| **5. I am cautious about sharing personal information on social media** | 0.247* |
| **6. I have received support and encouragement from online communities or groups** | 0.668 |
| **7. Social media helps me discover new hobbies and interests** | 0.449 |
| **8. I have taken breaks from social media to improve my well-being** | 0.500 |
| **9. I am mindful of my screen time and try to balance it with other activities** | 0.563 |
| **10. I have used social media to seek advice or guidance on personal issues** | 0.703 |
| **Dimension 2: Negative Digital Experiences** [R] (N = 6, α = 0.802, Mean inter-item r = 0.410) | |
| **11. I feel pressure to portray a perfect life on social media** | 0.498 |
| **12. Some people make fun of me on social media which affects my well-being** | 0.521 |
| **13. I find it difficult to disconnect from social media, even when I want to** | 0.662 |
| **14. Comparing myself to others on social media makes me feel inadequate** | 0.376 |
| **15. I often feel overwhelmed by the constant stream of information on social media** | 0.640 |
| **16. Social media can be a source of stress and anxiety for me** | 0.704 |

*[R] = item reverse scored prior to analysis. * item-total correlation below the 0.30 threshold*

***Table S6b. Inter-Item Correlation Matrices — Digital Well-being & Literacy***

| **Dimension 1: Positive Digital Engagement and Self-Regulation** | | | | | | | | | | |
| --- | --- | --- | --- | --- | --- | --- | --- | --- | --- | --- |
|  | **1** | **2** | **3** | **4** | **5** | **6** | **7** | **8** | **9** | **10** |
| **1. Social media helps me stay connected with my friends and family** | — | 0.279 | 0.371 | 0.503 | 0.028 | 0.071 | 0.141 | -0.161 | -0.341 | 0.306 |
| **2. Social media has a positive impact on my self-esteem** | 0.279 | — | 0.583 | 0.138 | 0.004 | 0.397 | 0.153 | 0.378 | 0.131 | 0.303 |
| **3. Social media provides a platform for me to express my creativity and interests** | 0.371 | 0.583 | — | 0.184 | 0.101 | 0.615 | 0.047 | 0.292 | 0.250 | 0.677 |
| **4. Social media is a useful tool for learning and accessing information** | 0.503 | 0.138 | 0.184 | — | -0.073 | -0.069 | 0.288 | -0.179 | -0.121 | 0.199 |
| **5. I am cautious about sharing personal information on social media** | 0.028 | 0.004 | 0.101 | -0.073 | — | 0.036 | 0.387 | 0.101 | 0.338 | 0.350 |
| **6. I have received support and encouragement from online communities or groups** | 0.071 | 0.397 | 0.615 | -0.069 | 0.036 | — | 0.188 | 0.667 | 0.563 | 0.604 |
| **7. Social media helps me discover new hobbies and interests** | 0.141 | 0.153 | 0.047 | 0.288 | 0.387 | 0.188 | — | 0.336 | 0.535 | 0.292 |
| **8. I have taken breaks from social media to improve my well-being** | -0.161 | 0.378 | 0.292 | -0.179 | 0.101 | 0.667 | 0.336 | — | 0.631 | 0.321 |
| **9. I am mindful of my screen time and try to balance it with other activities** | -0.341 | 0.131 | 0.250 | -0.121 | 0.338 | 0.563 | 0.535 | 0.631 | — | 0.537 |
| **10. I have used social media to seek advice or guidance on personal issues** | 0.306 | 0.303 | 0.677 | 0.199 | 0.350 | 0.604 | 0.292 | 0.321 | 0.537 | — |

| **Dimension 2: Negative Digital Experiences** | | | | | | |
| --- | --- | --- | --- | --- | --- | --- |
|  | **11** | **12** | **13** | **14** | **15** | **16** |
| **11. I feel pressure to portray a perfect life on social media** | — | 0.401 | 0.216 | 0.283 | 0.494 | 0.478 |
| **12. Some people make fun of me on social media which affects my well-being** | 0.401 | — | 0.413 | 0.241 | 0.430 | 0.448 |
| **13. I find it difficult to disconnect from social media, even when I want to** | 0.216 | 0.413 | — | 0.506 | 0.482 | 0.668 |
| **14. Comparing myself to others on social media makes me feel inadequate** | 0.283 | 0.241 | 0.506 | — | 0.190 | 0.179 |
| **15. I often feel overwhelmed by the constant stream of information on social media** | 0.494 | 0.430 | 0.482 | 0.190 | — | 0.723 |
| **16. Social media can be a source of stress and anxiety for me** | 0.478 | 0.448 | 0.668 | 0.179 | 0.723 | — |

# **Supplementary results table**

**Table S7. Mental health and well-being drivers among young Moroccans (*n* = 1182)**

| **Variables** | **Distribution N (%)** | | | | |
| --- | --- | --- | --- | --- | --- |
|  | **Extremely important** | **Very important** | **Moderately important** | **Slightly important** | **Not important at all** |
| **Good health and optimum nutrition** | | | | | |
| Having a varied and balanced diet | 341 (28.8) | 469 (39.6) | 227 (19.2) | 85 (7.2) | 61 (5.2) |
| Sleeping well at night | 548 (46.3) | 425 (35.9) | 133 (11.2) | 37 (3.1) | 40 (3.4) |
| Practicing in sports and exercise | 319 (27.0) | 463 (39.1) | 234 (19.8) | 94 (7.9) | 73 (6.2) |
| Being satisfied with the way I look | 418 (35.3) | 468 (39.6) | 176 (14.9) | 64 (5.4) | 57 (4.8) |
| Being physically healthy | 706 (59.7) | 365 (30.9) | 61 (5.2) | 18 (1.5) | 33 (2.8) |
| Having access to information about health | 308 (26.0) | 494 (41.8) | 240 (20.3) | 83 (7.0) | 58 (4.9) |
| Having access to quality health services | 628 (53.1) | 374 (31.6) | 107 (9.0) | 30 (2.5) | 44 (3.7) |
| Having access to green spaces | 472 (39.9) | 442 (37.4) | 167 (14.1) | 60 (5.1) | 42 (3.6) |
| Breathing clean air | 664 (56.5) | 359 (30.5) | 94 (8.0) | 28 (2.4) | 31 (2.6) |
| **Connectedness, positive values, and contribution to society** | | | | | |
| Having a good relationship with my parents and family | 896 (75.7) | 212 (17.9) | 39 (3.3) | 14 (1.2) | 22 (1.9) |
| Having a positive relationship with my teachers/ professors/ employer | 411 (34.7) | 483 (40.8) | 199 (16.8) | 49 (4.1) | 41 (3.5) |
| Having a positive relationship with my peers/ colleagues | 297 (25.1) | 503 (42.5) | 267 (22.6) | 73 (6.2) | 43 (3.6) |
| Feeling a sense of belonging in my community, school or work environment | 396 (33.5) | 512 (43.3) | 172 (14.5) | 54 (4.6) | 49 (4.1) |
| Having an adult person in my life who I can trust | 390 (33.0) | 383 (32.4) | 230 (19.4) | 95 (8.0) | 85 (7.2) |
| Feeling accepted, respected and valued by others | 435 (36.8) | 424 (35.8) | 202 (17.1) | 65 (5.5) | 57 (4.8) |
| Participating in social and cultural activities in the community or at school /university or work | 335 (28.3) | 390 (33.0) | 264 (22.3) | 118 (10.0) | 76 (6.4) |
| Having the chance to participate in decision-making and having my ideas valued and respected. | 472 (39.9) | 442 (37.4) | 171 (14.5) | 51 (4.3) | 47 (4.0) |
| Getting the chance to learn about empathy, make friends, and become more understanding | 354 (29.9) | 461 (39.0) | 227 (19.2) | 85 (7.2) | 56 (4.7) |
| **Safety and a supportive environment** | | | | | |
| Not being exposed to violence (including bullying, online harassment, physical, sexual, verbal abuse and emotional violence) | 704 (59.5) | 301 (25.4) | 75 (6.3) | 47 (4.0) | 56 (4.7) |
| Feeling safe in my daily life, whether at home, in my neighborhood, online, or at school or work. | 765 (64.7) | 302 (25.5) | 59 (5.0) | 26 (2.2) | 31 (2.6) |
| Being treated like others and without discrimination | 597 (50.5) | 368 (31.1) | 118 (10.0) | 46 (3.9) | 54 (4.6) |
| Feeling safe to express myself and be who I am | 630 (53.3) | 388 (32.8) | 93 (7.9) | 29 (2.5) | 43 (3.6) |
| Having essential needs such as food, water, a place to live, warmth, clothing, and feeling safe and secure | 828 (70) | 252 (21.3) | 54 (4.6) | 20 (1.7) | 29 (2.5) |
| My personal information is protected and is not shared without my permission | 754 (63.7) | 295 (24.9) | 74 (6.3) | 25 (2.1) | 35 (3.0) |
| Having access to leisure activities and personal development opportunities | 447 (37.8) | 449 (38.0) | 174 (14.7) | 68 (5.7) | 45 (3.8) |
| **Learning, competence, education, skills, and employability** | | | | | |
| Getting to go to school and having chances to keep learning, whether in a classroom or through other kinds of learning | 643 (54.4) | 385 (32.5) | 94 (7.9) | 26 (2.2) | 35 (3.0) |
| Getting help to stay motivated and keep learning, | 436 (36.9) | 468 (39.6) | 173 (14.6) | 62 (5.2) | 44 (3.7) |
| Having chances to build the tools and skills to succeed | 552 (46.7) | 473 (40.0) | 103 (8.7) | 17 (1.4) | 38 (3.2) |
| Having self-confidence and feeling that I can do things well | 736 (62.2) | 333 (28.1) | 58 (4.9) | 17 (1.4) | 39 (3.3) |
| Learning practical skills for work | 614 (51.9) | 410 (34.7) | 96 (8.1) | 30 (2.5) | 33 (2.8) |
| Working in jobs and businesses appropriate for my age | 481 (40.7) | 407 (34.4) | 180 (15.2) | 53 (4.5) | 62 (5.2) |
| Being satisfied with my learning and skills | 609 (51.5) | 431 (36.4) | 84 (7.1) | 28 (2.4) | 31 (2.6) |
| Believing in myself and my ability to reach my learning goals | 708 (59.8) | 356 (30.1) | 54 (4.6) | 26 (2.2) | 39 (3.3) |
| **Agency and resilience** | | | | | |
| Feeling independent and capable of making my own decisions | 703 (59.4) | 370 (31.3) | 71 (6.0) | 11 (0.9) | 28 (2.4) |
| Feeling empowered to accomplish things and having confidence in myself (whether with friends, family, or in decision-making) | 660 (55.8) | 406 (34.3) | 74 (6.3) | 14 (1.2) | 29 (2.5) |
| Having hope and optimism towards the future | 651 (55.0) | 407 (34.4) | 68 (5.7) | 24 (2.0) | 33 (2.8) |
| Having a sense of purpose in my life | 810 (68.5) | 263 (22.2) | 62 (5.2) | 14 (1.2) | 34 (2.9) |
| Having opportunities to develop the ability to handle the challenges in life both now and in the future | 622 (52.6) | 434 (36.7) | 78 (6.6) | 18 (1.5) | 31 (2.6) |
| Having chances to reach my full potential now and later in life. | 672 (56.8) | 379 (32) | 83 (7.0) | 17 (1.4) | 32 (2.7) |
| **Digital well-being and literacy** | **Completely agree** | **Agree** | **Undecided** | **Disagree** | **Strongly disagree** |
| **Dimension 1: Positive Digital Engagement and Self-Regulation** | | | | | |
| Social media helps me stay connected with my friends and family. | 446 (37.7) | 570 (48.2) | 93 (7.9) | 47 (4.0) | 27 (2.3) |
| Social media has a positive impact on my self-esteem. | 131 (11.1) | 306 (25.9) | 340 (28.7) | 228 (19.3) | 178 (15.0) |
| Social media provides a platform for me to express my creativity and interests. | 244 (20.6) | 465 (39.3) | 249 (21.0) | 131 (11.1) | 94 (7.9) |
| Social media is a useful tool for learning and accessing information. | 450 (38.0) | 525 (44.4) | 128 (10.8) | 54 (4.6) | 26 (2.2) |
| I am cautious about sharing personal information on social media. | 514 (43.4) | 397 (33.6) | 149 (12.6) | 70 (5.9) | 53 (4.5) |
| I have received support and encouragement from online communities or groups. | 215 (18.2) | 406 (34.3) | 271 (22.9) | 159 (13.4) | 132 (11.2) |
| Social media helps me discover new hobbies and interests. | 382 (32.3) | 539 (45.6) | 168 (14.2) | 47 (4.0) | 47 (4.0) |
| I have taken breaks from social media to improve my well-being. | 402 (34.0) | 389 (32.9) | 178 (15.0) | 119 (10.1) | 95 (8.0) |
| I am mindful of my screen time and try to balance it with other activities. | 353 (29.8) | 443 (37.4) | 210 (17.8) | 114 (9.6) | 63 (5.3) |
| I have used social media to seek advice or guidance on personal issues. | 352 (29.8) | 379 (32.0) | 192 (16.2) | 115 (9.7) | 145 (12.3) |
| **Dimension 2: Negative Digital Experiences*** | | | | | |
| I feel pressure to portray a perfect life on social media. | 147 (12.4) | 222 (18.8) | 213 (18.0) | 281 (23.8) | 320 (27.0) |
| Some people make fun of me on social media platforms which affects my well-being. | 109 (9.2) | 175 (14.8) | 206 (17.4) | 232 (19.6) | 461 (39.0) |
| I find it difficult to disconnect from social media, even when I want to. | 235 (19.9) | 305 (25.8) | 243 (20.5) | 217 (18.3) | 183 (15.5) |
| Comparing myself to others on social media makes me feel inadequate. | 203 (17.2) | 275 (23.2) | 214 (18.1) | 185 (15.6) | 306 (25.9) |
| I often feel overwhelmed by the constant stream of information on social media. | 242 (20.5) | 407 (34.4) | 292 (24.7) | 135 (11.4) | 107 (9.0) |
| Social media can be a source of stress and anxiety for me. | 374 (31.6) | 386 (32.6) | 204 (17.2) | 129 (10.9) | 90 (7.6) |

**items in Dimension 2 were reverse scored prior to reliability analysis*
