# Supplementary material for: Drivers of youth mental health and wellbeing: a large-scale cross-sectional study in Morocco
Source: BMJ Open. 2026 Jun 9;16(6):e110683. doi: 10.1136/bmjopen-2025-110683 (PMC13264952; doi:10.1136/bmjopen-2025-110683)
Supplement: Supplementary file 3 [file bmjopen-2025-110683.reviewer_comments.pdf]

PEER REVIEW HISTORY

BMJ Open publishes all reviews undertaken for accepted manuscripts. Reviewers are asked to complete a checklist review form and are provided with free text boxes to elaborate on their assessment. These free text comments are reproduced below.

ARTICLE DETAILS

Title (Provisional)

Drivers of youth mental health and wellbeing: A large-scale cross-sectional study in Morocco

Authors

Bnimoussa, Jihad; Bouaddi, Oumnia; Elbadisy, Imad; Khalis, Mohamed

---

VERSION 1 - REVIEW

**Reviewer**	**1**
**Name**	**Swenson, Cynthia Cupit**
**Affiliation**	**Medical University of South Carolina, Psychiatry**
**Date**	**09-Feb-2026**
**COI**	**None**

This study, implemented across 13 regions in Morocco, had the aim of assessing drivers of mental health well-being among young people ages 18 to 24 in a country that has high rates of mental health difficulties and substance use and limited mental health professionals. In addition research on drivers of youth mental health and well-being in Morocco is limited. As such, the study fills a gap in the literature.

A strength of the study is that well-being is considered across psychological, social, environmental, cognitive, and biological systems.

Page 4, line 92 – use of the term sick. This term implies that mental health challenges are a disease or illness but the focus of this study implies mental health challenges are multi-determined and so the notion of sickness does not fit. A more appropriate word should be considered such as “conditions that led to their mental health challenges.

Page 4, line 101 – the word dementia seems out of a place in a study of youth. If the au views it as an appropriate term for this age range, it should be explained how this term fits with youth.

Page 6. It is noted that the Adolescent Wellbeing Framework was validated with a group of 20 young Moroccans. Please present data on preliminary reliability and validity even though the N was small.

The study is described as an online survey that was distributed through Whatsapp. Please provide more information on how informed consent was attained and the role of the person attaining the informed consent. Please explain how the participant contact information was attained by the research team as the distribution was through youth serving organizations.

Please expound on how the youth serving organizations made Whatsapp contact with potential participants to recruit once they had contact information.

Also, Whatsapp is widely used in LMIC but may not be a protected platform. Please explain how confidential personal health information was protected. It is noted that the study was approved by an ethics committee so it's assumed that confidentiality measures were taken care of but it would help to know how.

With regard to generalizability, the au notes the issue of use of Whatsapp may leave out youth who have economic disadvantages that prevent them from owning or using devices onto which Whatsapp can be downloaded. In addition, au notes that rural areas were highly underrepresented. It should also be noted that males were highly underrepresented and individuals with high educational attainment were overrepresented in the study.

Au should note whether there are any cultural factors that may impact use of a Likert scale, such as culturally do individuals tend to stay middle of the road on responses with not using what may be viewed as, extremes.

The sample size (1182) is a strength of the study. Please note on page 11, line 224 whether it should read n=1182 or N=1182.

Please explain why analyses by age were grouped < 20 and >20.

Please indicate whether au has data to describe economic status of families and if au has data, please analyze.

With 1182 participants, the au should be able to assess reliability (at least internal consistency). Please do so or explain why not. These data are very important to understanding the reliability of the measure.

The study revealed important drivers of mental health and well-being among Moroccan youth, primarily female, highly educated and in an urban setting. It's very helpful that au lists the top 10 drivers in a table. Thank you.

The study is the first of its kind. The outcomes of the study are informative and could assist individuals that work with young people in meeting their needs for positive mental health and wellness. Considerations for answers to the reviewer questions and additions and edits are indicated.

**Reviewer**	**2**
**Name**	**Cusworth Walker, S**

Affiliation **University of Washington School of Medicine, Psychiatry and Behavioral Scieences**

Date **14-Mar-2026**

COI **None**

Thank you for the opportunity to review this paper. The authors present the results of an impressive, country-wide effort to capture youth perceptions of subjective well-being in order to assess primary drivers of mental health. The approach and the presentation of findings include notable strengths including youth-engaged research processes, strong participant response, and survey development in a novel area (youth well-being). The presentation of results, discussion and limitation section could be improved to better highlight the key findings as an analysis of priorities likely to inform future policy or intervention initiatives.

The introduction is well-organized and researched. The authors argue that large-scale studies are needed to better understand the drivers of youth well-being in the country being studied (Morocco) in order to craft responsive policies. This is a strong argument that needs better development in the presentation of results and discussion.

The methods include good conceptual grounding for survey development and construction and a generally clear description of research procedures. However, I am not clear what the authors mean when describing sampling methods as randomly stratified by socio demographic backgrounds in addition to noting the use of convenience sampling. My understanding is that these would be conflicting methods (random stratification suggests purposive sampling). The description of how youth research liaisons were trained suggests they disseminated the survey through their existing networks (e.g., convenience sampling vs. random stratification).

Descriptions of how the measure of well-being was created are scattered throughout the paper and would benefit from being collected into one section (e.g., scale construction) in addition to presenting more information on survey psychometrics, including inter-item correlations and subscale score reliability, given the novelty of the scale. I would suggest this information be presented in the Methods, leaving the Results section for comparisons among subscales and in group comparison (region, age, gender).

The Results section would be improved by a clearer description of when specific statistical analyses were used. Currently, the manuscript includes p values without the accompanying statistical tests in the narrative. Similarly, statistical tests and results are not included in the tables (where only percentages are reported). Given the study is aiming to inform country policies, I also expected to see results reported by region. The results (and table) would benefit from analysis beyond descriptive statistics of survey item responses as it is difficult to draw any conclusions from the raw data. I was not clear how the top ten drivers of mental

health were related to the measure scale and subscales as this table seemed to introduce new constructs.

Some of the conceptual confusion in the Results spills over into the Discussion section. As presented, the survey appears to be measuring how youth rate/scale the drivers of mental health, not their prevalence. In other words, youth could rate family relationship as a critical factor in mental health and that would not indicate whether that factor was low or high in the population. If the authors measured prevalence of need, this should be more clear throughout the paper. Correspondingly, the authors should be careful to limit their discussion and conclusions to their findings. In line 333 (pg. 17) the authors assert that youth rated family relationships higher than poverty as a driver of well-being but the results include a table without accompanying statistics or rationale for the rating order, nor are the results of the rating order assessed by sociodemographic factors of respondents.

Overall, the paper is positioned to make a good contribution to the literature on youth-informed agenda setting for youth wellbeing policy if the authors can address the questions raised in this review.

VERSION 1 - AUTHOR RESPONSE

Reviewer: 1
Dr. Cynthia Cupit Swenson, Medical University of South Carolina

Comments to the Author:

This study, implemented across 13 regions in Morocco, had the aim of assessing drivers of mental health well-being among young people ages 18 to 24 in a country that has high rates of mental health difficulties and substance use and limited mental health professionals. In addition research on drivers of youth mental health and well-being in Morocco is limited. As such, the study fills a gap in the literature. A strength of the study is that well-being is considered across psychological, social, environmental, cognitive, and biological systems.

Response: Thank you for your positive feedback.

1. Page 4, line 92 – use of the term sick. This term implies that mental health challenges are a disease or illness but the focus of this study implies mental health challenges are multi-determined and so the notion of sickness does not fit. A more appropriate word should be considered such as "conditions that led to their mental health challenges.

Response: Thank you for flagging this error, as the use of disorder-based language does not align with the values and orientation of the study, and the use of that term was not intentional. The oversight has been corrected.

"Relieving the burden of mental health conditions requires both treating young people as well as addressing the conditions that led to their mental health challenges in the first place (6)."

2. Page 4, line 101 – the word dementia seems out of a place in a study of youth. If the au views it as an appropriate term for this age range, it should be explained how this term fits with youth.

Response: Thank you for flagging this. The inclusion of the word was emergent from the study referenced by that line on social determinants and the specific disorders they impact. The sentence has been edited to retain only the information pertinent to our study.

3. Page 6. It is noted that the Adolescent Wellbeing Framework was validated with a group of 20 young Moroccans. Please present data on preliminary reliability and validity even though the N was small.

Response: Thank you for your question. The survey, developed by the authors for the study, was based on the existing Adolescent Wellbeing Framework and was validated with a small group of participants prior to the start of the study. The data collection section has been updated to include the requested data.

"To assess preliminary internal consistency reliability, Cronbach's alpha coefficients and corrected item-total correlations were computed for each subscale among the 20 Moroccan young people who participated in the pilot validation phase. All five AWF subscales demonstrated excellent internal consistency: Good Health and Optimum Nutrition ($\alpha = 0.938$), Connectedness, Positive Values and Contribution to Society ($\alpha = 0.952$), Safety and Supportive Environment ($\alpha = 0.956$), Learning, Competence, Education and Employability ($\alpha = 0.963$), and Agency and Resilience ($\alpha = 0.952$). Corrected item-total correlations across these five subscales ranged from 0.558 to 0.956, with all items exceeding the conventional threshold of 0.30. For the Digital Well-being domain, both dimensions demonstrated acceptable to good internal consistency (Positive Digital Engagement and Self-Regulation: $\alpha = 0.781$; Negative Digital Experiences: $\alpha = 0.802$). All item-total correlations in the Negative Digital Experiences dimension exceeded the conventional threshold of 0.30 (range: 0.376–0.704). Three items in the Positive Digital Engagement dimension yielded correlations below 0.30, likely reflecting ceiling effects given the small pilot sample size rather than item misfit. Full item-level statistics are reported in Supplementary Tables S2–S6."

4. The study is described as an online survey that was distributed through Whatsapp. Please provide more information on how informed consent was attained and the role of the person attaining the informed consent.

Response: Thank you for your pertinent query. The following clarification has been added to the study design and setting section of the manuscript.

"The online survey included two questions before the start of the questionnaire pertaining to consent. Participants indicated if they were between 18 to 24 years old and whether or not they consent to participating in the study. The survey was anonymous and did not collect names of participants. While the online survey was spread through a network of individuals through WhatsApp, the authors did not have any direct contact with participants."

5. Please explain how the participant contact information was attained by the research team as the distribution was through youth serving organizations.

Response: Thank you for requesting clarification. The link to the anonymous survey was distributed through a network of youth serving organizations to their own pre-existing communities across the 12 regions of Morocco with the aim of reaching participants between the ages of 18 and 24. A clarification regarding contact has been added to the Study design and setting section of the manuscript.

"While the online survey was spread through a network of individuals through WhatsApp, the authors did not have any direct contact with participants."

6. Please expound on how the youth-serving organizations made Whatsapp contact with potential participants to recruit once they had contact information.

Response: Thank you for your comment. We have clarified the recruitment process in the revised manuscript. The following text has been added to the Study Design and Setting section:

"Youth serving organizations are grassroots organizations local to different regions in Morocco. They operate within their communities through the regular organization of community events, workshops, and other youth-benefiting activities. As a result, they have an existing contact list of young people who receive announcements of these activities and had voluntarily signed up to be informed of the activities of the organization. As most of these notices are shared through Whatsapp, not an email newsletter as is more common in Global North settings, the youth serving organizations made contact with potential participants through Whatsapp."

7. Also, Whatsapp is widely used in LMIC but may not be a protected platform. Please explain how confidential personal health information was protected. It is noted that the study was approved by an ethics committee so it's assumed that confidentiality measures were taken care of but it would help to know how.

Response: Thank you for your comment. We have explained and clarified confidentiality issues in the revised manuscript in the Study Design and Setting Section:

"There was no sharing of confidential information directly through Whatsapp. Whatsapp as an accessible form of communication has double encryption which is why local grassroots organizations use it to connect with their communities. The electronic survey that was shared was created and shared through a secure institutional server with no third party access to the raw data. The survey was analyzed in a de-identified manner and did not collect the names of participants."

8. With regard to generalizability, the au notes the issue of use of Whatsapp may leave out youth who have economic disadvantages that prevent them from owning or using devices onto which Whatsapp can be downloaded. In addition, au notes that rural areas were highly underrepresented. It should also be noted that males were highly underrepresented and individuals with high educational attainment were overrepresented in the study.

Response: Thank you for this important comment. We have clarified our explanation of our sampling method. We have also updated the limitations of the study to include clearer referencing of the demographic limitations.

"The participants were recruited through non-probabilistic, convenience sampling and in order to include young people representing the 12 regions of Morocco sample size was allocated per regions and sub-regions, with specified sociodemographic characteristics. Therefore, there was a skew towards young people with access to digital devices who could be reached through this sampling method thus underrepresenting rural participants. The data collection methodology was a digital survey, rather than an in-person data collection method, which resulted in an overrepresentation of participants with higher education. As outreach to potential participants was done through youth serving organizations who sent out the electronic survey to their local community of young people who participate in the organization's activities, the gender of study participants skewed to primarily female participants as they tend to be overrepresented in activity participation. Therefore, this study, while reaching a larger number of participants than existing studies, is not representative of all Moroccan youth with differing abilities, needs, or socio-economic backgrounds thus limiting the national generalizability of the study's results."

9. Au should note whether there are any cultural factors that may impact use of a Likert scale, such as culturally do individuals tend to stay middle of the road on responses with not using what may be viewed as extremes.

Response: Thank you for your comment. The survey was anonymized to mitigate social approval bias. The authors' previous experience conducting research in the Moroccan context informs our understanding that this "tendency to stay middle of the road on responses" is not an issue. Culturally, Moroccans tend to be vocal about their opinions within the realm of relevant topics to this study.

10. The sample size (1182) is a strength of the study. Please note on page 11, line 224 whether it should read n=1182 or N=1182.

Response: Thank you for your comment. We have fixed this formatting error.

11. Please explain why analyses by age were grouped < 20 and >20.

Response: Thank you for your comment. The 20 figure is the median, this has been clarified in the revised manuscript:

"The mean age of participants was 20.5 years old and the median was 20. "

12. Please indicate whether au has data to describe economic status of families and if au has data, please analyze.

Response: Thank you for your interest in this additional data. This data was not collected as part of our study. The closest proxy for economic status of families in our data set would be professional status and level of education

13. With 1182 participants, the au should be able to assess reliability (at least internal consistency). Please do so or explain why not. These data are very important to understanding the reliability of the measure.

Response: Thank you for this important comment. We have now conducted internal consistency analysis on the pilot validation sample (N = 20) and report Cronbach's alpha coefficients for all subscales. Regarding the Digital Well-being and Literacy scale specifically, we took this opportunity to revisit its structure in light of the reviewer's feedback. We have reorganized this domain into two dimensions (theoretically driven). The following text has been added to the data collection section, and the tables have been updated accordingly in the manuscript:

“Consistent with established evidence that positive and negative dimensions of digital well-being represent empirically distinct constructs, the Digital Well-being and Literacy domain was organized into two dimensions: Positive Digital Engagement and Self-Regulation (10 items measuring beneficial social media use, creative expression, and intentional self-regulation behaviors) and Negative Digital Experiences (6 items measuring social comparison, cyberbullying, problematic use, and stress).

To assess preliminary internal consistency reliability, Cronbach's alpha coefficients and corrected item-total correlations were computed for each subscale among the 20 Moroccan young people who participated in the pilot validation phase. All five AWF subscales demonstrated excellent internal consistency: Good Health and Optimum Nutrition (α = 0.938), Connectedness, Positive Values and Contribution to Society (α = 0.952), Safety and Supportive Environment (α = 0.956), Learning, Competence, Education and Employability (α = 0.963), and Agency and Resilience (α = 0.952). Corrected item-total correlations across these five subscales ranged from 0.558 to 0.956, with all items exceeding the conventional threshold of 0.30. For the Digital Well-being domain, both dimensions demonstrated acceptable to good internal consistency (Positive Digital Engagement and Self-Regulation: α = 0.781; Negative Digital Experiences: α = 0.802). All item-total correlations in the Negative Digital Experiences dimension exceeded the conventional threshold of 0.30 (range: 0.376–0.704). Three items in the Positive Digital Engagement dimension yielded correlations below 0.30, likely reflecting ceiling effects given the small pilot sample size rather than item misfit. Full item-level statistics are reported in Supplementary Tables S2–S6."

14. The study revealed important drivers of mental health and well-being among Moroccan youth, primarily female, highly educated and in an urban setting. It’s very helpful that au lists the top 10 drivers in a table. Thank you. The study is the first of its kind. The outcomes of the study are informative and could assist individuals that work with young people in meeting their needs for positive mental health and wellness. Considerations for answers to the reviewer questions and additions and edits are indicated.

Response: Thank you for the positive feedback.

Reviewer: 2
Dr. S Cusworth Walker, University of Washington School of Medicine

Comments to the Author:
1. Thank you for the opportunity to review this paper. The authors present the results of an impressive, country-wide effort to capture youth perceptions of subjective well-being in order to assess primary drivers of mental health. The approach and the presentation of findings include notable strengths, including youth-engaged research processes, strong participant response, and survey development in a novel area (youth well-being).

The introduction is well-organized and researched. The authors argue that large-scale studies are needed to better understand the drivers of youth well-being in the country being studied (Morocco) in order to craft responsive policies. This is a strong argument that needs better development in the presentation of results and discussion.
The presentation of results, discussion, and limitation section could be improved to better highlight the key findings as an analysis of priorities likely to inform future policy or intervention initiatives.

Response: Thank you for your encouraging comment. We have edited the relevant sections to clarify the role that understanding the priority drivers of wellbeing from the perspective of Moroccan youth can play in future policy and intervention design:

"The study centers the perspectives of young people and their subjective evaluations of the drivers they consider to be a priority to their own wellbeing. It can provide important guidance for further research to better understand drivers of wellbeing, policy regarding protections for youth wellbeing, and practice for intervention and program design to promote wellbeing. Data on wellbeing is not systematically collected in Morocco therefore it is likely to be fragmented and focused on the prevalence of psychiatric disorders. However, the Ministry of Health in Morocco is developing a multi-sectoral strategy and this study is in line with the current efforts to inform youth related aspects of this strategy. This study can help inform the training of counselors and mental health professionals (32) by providing a list of priority areas for psychoeducation and prevention programs in schools and community settings. It can also expand the conversation in Morocco beyond mental illnesses to focus reforms on addressing social and environmental determinants of mental health and wellbeing with local guidance on drivers of mental health to help address inequities which impact young people's mental health. It can be used to guide youth programming, especially in providing context-relevant interventions that support the development of positive relationships with parents and family members such as in the Pathways Triple P (PTP) program, that build a sense of purpose, self-efficacy, and capacity (33). It can also inform policy that seeks to address inequities through the reduction of poverty and creating safer environments for young people in schools, their communities, and at home by opening new avenues of institutional collaboration through the lens of youth mental health. Having the perspectives of Moroccan youth from across 12 regions speaking to the drivers of mental health from their perspective can help enhance the inclusion of youth lived experiences (young people's subjective evaluations and expressions of their experiences and perspectives) in policy and practice and encourage further participatory research."

The methods include good conceptual grounding for survey development and construction and a generally clear description of research procedures. However, I am not clear what the authors mean when describing sampling methods as randomly stratified by socio-

demographic backgrounds in addition to noting the use of convenience sampling. My understanding is that these would be conflicting methods (random stratification suggests purposive sampling). The description of how youth research liaisons were trained suggests they disseminated the survey through their existing networks (e.g., convenience sampling vs. random stratification).

Response: Thank you for your comment. We have mistakenly mentioned random sampling. It was an omission on our part. We have corrected this in the revised manuscript:

"The study used non-probabilistic, convenience sampling; however, in order to include young people representing the 12 regions of Morocco, sample size was allocated per regions and sub-regions, with sociodemographic characteristics such as place of residence, sex and education status being considered in participant outreach."

2. Descriptions of how the measure of well-being was created are scattered throughout the paper and would benefit from being collected into one section (e.g., scale construction) in addition to presenting more information on survey psychometrics, including inter-item correlations and subscale score reliability, given the novelty of the scale. I would suggest this information be presented in the Methods, leaving the Results section for comparisons among subscales and in group comparison (region, age, gender).

Response: Thank you for your comment. We have added a section under data collection that clearly explains the construction of the scale and its psychometric testing.

"To assess preliminary internal consistency reliability, Cronbach's alpha coefficients and corrected item-total correlations were computed for each subscale among the 20 Moroccan young people who participated in the pilot validation phase. All five AWF subscales demonstrated excellent internal consistency: Good Health and Optimum Nutrition (α = 0.938), Connectedness, Positive Values and Contribution to Society (α = 0.952), Safety and Supportive Environment (α = 0.956), Learning, Competence, Education and Employability (α = 0.963), and Agency and Resilience (α = 0.952). Corrected item-total correlations across these five subscales ranged from 0.558 to 0.956, with all items exceeding the conventional threshold of 0.30. For the Digital Well-being domain, both dimensions demonstrated acceptable to good internal consistency (Positive Digital Engagement and Self-Regulation: α = 0.781; Negative Digital Experiences: α = 0.802). All item-total correlations in the Negative Digital Experiences dimension exceeded the conventional threshold of 0.30 (range: 0.376–0.704). Three items in the Positive Digital Engagement dimension yielded correlations below 0.30, likely reflecting ceiling effects given the small pilot sample size rather than item misfit. Full item-level statistics are reported in Supplementary Tables S2–S6."

The Results section would be improved by a clearer description of when specific statistical analyses were used. Currently, the manuscript includes p values without the accompanying statistical tests in the narrative. Similarly, statistical tests and results are not included in the tables (where only percentages are reported). Given the study is aiming to inform country policies, I also expected to see results reported by region. The results (and table) would benefit from analysis beyond descriptive statistics of survey item responses as it is difficult to draw any conclusions from the raw data.

Response: Thank you for your comment. We have specified in the data analysis section the tests used. We have also added an analysis across regions to understand whether there are any differences across regions (See table 2). The following text has been added to the revised manuscript:

Data collection:
"In addition, we used ANOVA One-Way to examine any differences exist between the region across the six well-being domains. "

Results:
"There were statistically significant differences between the regions across five out of the six well-being domains, with "Good health and optimum nutrition"n being the exception (p=0.119)"

I was not clear how the top ten drivers of mental health were related to the measure scale and subscales as this table seemed to introduce new constructs. Some of the conceptual confusion in the Results spills over into the Discussion section. As presented, the survey appears to be measuring how youth rate/scale the drivers of mental health, not their prevalence. In other words, youth could rate family relationship as a critical factor in mental health and that would not indicate whether that factor was low or high in the population. If the authors measured prevalence of need, this should be more clear throughout the paper. Correspondingly, the authors should be careful to limit their discussion and conclusions to their findings.

Response: Thank you for your important comment. The study, indeed, seeks to evaluate the priority drivers of wellbeing from the perspective of young Moroccans, not their prevalence. We have reviewed the paper to ensure that our discussion of the results is not misconstrued as indicating prevalence.

In line 333 (pg. 17) the authors assert that youth rated family relationships higher than poverty as a driver of well-being but the results include a table without accompanying

statistics or rationale for the rating order, nor are the results of the rating order assessed by sociodemographic factors of respondents.

Response: Thank you for your comment. We have updated the table that shows the rating order that participants provided for different determinants of wellbeing (see updated Box 1). Due to the limited data on sociodemographic factors of respondents, we did not want to insinuate generalizability of the data and in that regard necessary edits to ensure that have been made.

Overall, the paper is positioned to make a good contribution to the literature on youth-informed agenda setting for youth wellbeing policy if the authors can address the questions raised in this review.

Response: Thank you for your positive feedback.
